# How international organizations can support the development of cardio-oncology in the Latin American and the Caribbean region

**DOI:** 10.1093/ehjimp/qyae005

**Published:** 2024-02-14

**Authors:** Amalia Peix, Manuel Bazan, Jorge E Aguiar, Jesus Sanchez, Enrique Estrada, Aurelio Mendoza, Diana Paez

**Affiliations:** Nuclear Medicine Department, Institute of Cardiology and Cardiovascular Surgery, Havana, Cuba; Cardiooncology Section, Institute of Oncology and Radiobiology, Havana, Cuba; Nuclear Medicine Department, Institute of Cardiology and Cardiovascular Surgery, Havana, Cuba; Statistics Department, Institute of Cybernetics, Mathematics and Physics, Havana, Cuba; Nuclear Medicine and Diagnostic Imaging Section, Division of Human Health, Department of Nuclear Sciences and Applications, International Atomic Energy Agency, Vienna, Austria; Nuclear Medicine Department, National Cardiovascular Institute INCOR, Jr. Coronel Zegarra 417 Jesus Maria, Lima, Peru; Nuclear Medicine and Diagnostic Imaging Section, Division of Human Health, Department of Nuclear Sciences and Applications, International Atomic Energy Agency, Vienna, Austria

**Keywords:** cardio-oncology, cardiotoxicity, LAC, multimodality imaging

## Abstract

Health problems in the Latin American and the Caribbean (LAC) region are highly concentrated on non-communicable diseases, being cardiovascular diseases (CVDs) and cancer the leading causes of death. Different countries of the region are at different stages of development in addressing CVDs and cancer. Opportunities for training and continuing education in cardio-oncology, as well as active cardio-oncology groups, are primarily limited to large academic institutions in major metropolitan areas. In addition, the development of advanced imaging modalities in LAC faces challenges such as the high cost of equipment, a lack of equipment maintenance and service, as well as insufficient specific training for both imaging specialists and referring clinicians. To contribute to the implementation of actionable strategies ensuring equitable access to care for all, international organizations, such as the International Atomic Energy Agency (IAEA), offer support for the regional development of health projects that address educational needs. In this context, a new IAEA regional cooperation project for LAC titled ‘Strengthening of regional capacities on the use of Nuclear Medicine techniques in a Cardio-oncology multimodality approach in patients with cancer’ will be developed during 2024–2025. The experience of some centres, as well as national experiences in certain countries of the region, that have been previously involved in other regional projects, can be leveraged for the benefit of the entire region. We present a proposed road map for cross-institutional/countries collaboration in the development of cardio-oncology in the LAC region, contributing to decreasing the barriers to the growth of the subspecialty.

Health problems in the Latin American and the Caribbean (LAC) region are highly concentrated on non-communicable diseases (NCDs). Increased life expectancy, with a delayed epidemiological transition from communicable to NCDs, as well as factors such as globalization, urbanization, increase of diabetes mellitus, obesity, and physical inactivity, contribute to cardiovascular diseases (CVDs) being the leading cause of death in the region: in 2019, 2.0 million people died from CVD.^1^ On the other hand, cancer is the second leading cause of mortality. In 2019, cancer caused 1.4 million deaths.^2^ Of all NCD deaths, 35% occurred prematurely in people aged 30 to 70, with cardiovascular diseases and cancer combined accounting for 65% of total premature deaths.^3^ Although the burden of NCDs is a global health issue, it is essential to recognize that it represents a particularly heavy load (both socially and economically) for low- and middle-income countries (LMICs). Thus, NCDs constitute a big hurdle to development and poverty mitigation in these LMICs and are considered as part of the United Nations sustainable development agenda.

In addition, COVID has influenced the progression of some NCDs, leading to a significant delay in the diagnosis and treatment of these NCDs, resulting in higher mortality rates and a considerable expenditure of resources in LMICs. In LAC, a 17% decrease of cardiac diagnostic procedures persisted with only a 79% recovery rate.^4–6^

The high burden of risk factors common for CVDs and cancer in LAC is shared by different countries of the region, but they are at different stages of development in addressing this problem. Cardio-oncology groups have been established in several countries, such as Argentina, Brazil, Chile, Costa Rica, Cuba, and Peru. However, the current number of groups is still insufficient and there is no regional approach addressing the need for their creation. A particular challenge is that opportunities for training and continuing education in cardio-oncology, as well as active cardio-oncology groups, are primarily limited to large academic institutions in major metropolitan areas.

It is important to point out that the value of the multimodality imaging approach has significantly increased in contemporary Cardiology. However, the development of advanced imaging modalities in LAC faces challenges such as the high cost of equipment, a lack of equipment maintenance and service, as well as insufficient specific training for both imaging specialists and referring clinicians. There are significant differences by countries in resources allocated to health. Countries with the highest number of scanners (CT, MRI, SPECT, and PET-CT) per million inhabitants are Argentina, Brazil, Chile, and Colombia, showing a substantial difference from the rest of the countries.^7^ As a result, all of these factors contribute to disparities in patient care.

Another important aspect to consider is the social determinants of health (SDOH), a term introduced by the World Health Organization in 2003. SDOH encompass the social conditions in which people are born, live, and work, providing a more inclusive view of how factors such as environment, geographic location, neighbourhoods, access to healthcare, education, nutrition, and socioeconomics play a critical role in morbidity and mortality behaviours.^8^ Clinicians should consider SDOH when treating patients, as health decision-makers should also incorporate them when establishing locally tailored health politics. This is particularly important in LMICs with limited resources.

To contribute to the implementation of actionable strategies ensuring equitable access to care for all, international organizations, such as the International Atomic Energy Agency (IAEA), offer support for the regional development of health projects that address educational needs. In this context, a new IAEA regional cooperation project for LAC titled ‘Strengthening of regional capacities on the use of Nuclear Medicine techniques in a Cardio-oncology multimodality approach in patients with cancer’ will be developed during 2024–2025. The experience of some centres, as well as national experiences in certain countries of the region, that have been previously involved in other regional projects, can be leveraged for the benefit of the entire region.

Available capabilities and existing physical infrastructure in counterpart centres will be utilized to build capacities and disseminate know-how, addressing regional challenges in the field through the application of multimodality imaging techniques for the diagnosis and risk stratification of patients with cardiotoxicity derived from oncologic treatments. This approach will particularly benefit countries with limited resources. The project’s outcomes will be sustained through an established network and regional scientific activities. This regional collaborative approach aims to identify existing national capacities that can contribute to strengthening regional capacities. Moreover, also it will enable the maximization of the impact of the allocated resources for the development of the various imaging techniques in Cardiology.

In addition, this project will facilitate the integration of imaging techniques into a diagnostic algorithm with a cost-effective approach. It involves the collaboration among experts in Cardiac Imaging, Cardiology, and Oncology from different countries in the region. As noted by Westwood *et al*.^9^ in a different context, a professional barrier created around the ‘specialty-based’ rather than ‘competence-based’ delivery of cardiac services (in particular for cardiac CT and MRI) has been one of the major limiting factors for the widespread use of essential diagnostic tests in many countries, including those of the LAC region. Therefore, this project also aims to provide training for all imaging specialists, including nuclear medicine specialists, radiologists, and cardiologists in a multimodality framework. This recognizes that cardiac imaging is crucial for advancing precision and individualized medicine.

Effective linkages between stakeholders and end users, including patients with coronary artery disease, cancer patients, as well as patients’ associations, have already been established during previous projects. This collaboration involved counterpart medical institutions from the participating countries, scientific medical societies at national, regional, and international levels, and IAEA support, and will be used again during this new project. The main results will be disseminated through social media to raise awareness of the importance of early diagnosis and risk stratification of cardiotoxicity and post-radiotherapy damage in oncologic patients. Focus should not be placed only on the diagnostic accuracy but also on the effectiveness in improving outcomes.

National counterparts of this regional project will be in charge of the organization of national training activities to disseminate the knowledge and know-how obtained through the project, with the help of national health authorities and related medical societies, as well as their application to the cardiovascular health of oncologic patients in the region. To ensure sustainability of project outputs and outcomes, national counterparts will establish a compromise with national health authorities that will conduct the project implementation, including the commitment of necessary resources, as well as its sustainability after project completion. Regional scientific associations of the region such as the Interamerican Society of Cardiology (SIAC) and national cardiology and oncology societies will be involved.

IAEA can also help in other areas through different kinds of projects, such as research support through coordinated research projects where centres from different countries across the world participate, building infrastructure through the support of national projects, and policy advocacy.

As a summary, *Figure 1* presents a proposed road map for cross-institutional/countries collaboration in the development of cardio-oncology in the LAC region, contributing to decreasing the barriers to the growth of the subspecialty.

**Figure 1:**
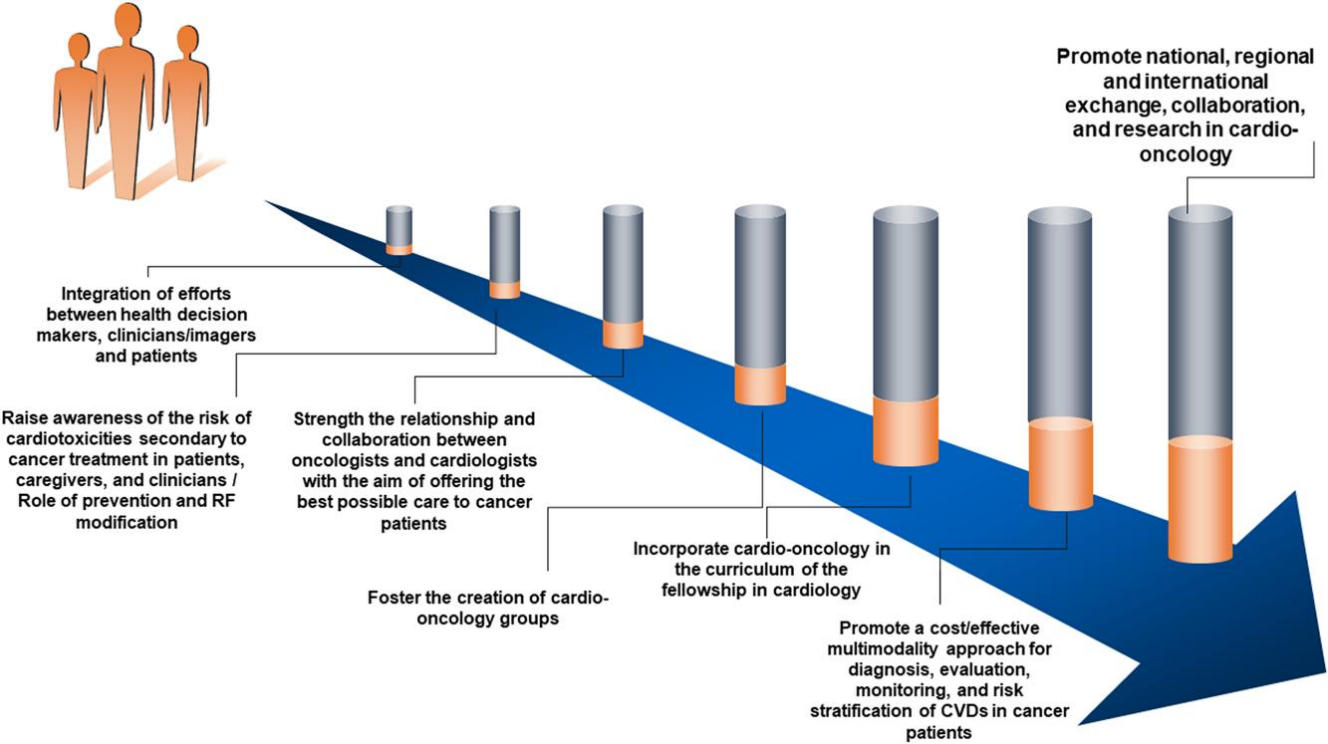
Proposed road map for cross-institutional/countries collaboration in the development of cardio-oncology in the LAC region. CVDs, cardiovascular diseases; RF, risk factor.

## Data Availability

No new data were generated or analysed in support of this research.
